# Expert Testimony

**Published:** 1891-02

**Authors:** Sidney B. Wright

**Affiliations:** Attorney at Law, Chattanooga, Tenn.


### EXPERT TESTIMONY.

BY SIDNEY B. WRIGHT, ATTORNEY AT LAW, CHATTANOOGA, TENN.

The word evidence in its legal acceptation includes all the means by which any alleged matter of fact, the truth of which is submitted to investigation, is established or disproved

The usual means of establishing or disproving matters of fact under inquiry are the statements made by witnesses in Court under a legal sanction—which statements are usually denominated testimony.

The mathematician can easily convince his pupil that the square or the hypothenuse of a right angled triangle is equal to the sum of the squares of the other two sides. But none save mathematical truths are susceptible of that high degree of evidence called demonstration—excluding all possibility of error.

The chemist shows his audience the make-up of an alloy by subjecting it to the crucible, and thus segregating it into its component elements.

The physician administers this powder or that potion because he is able to argue by induction that it is the thing, because for scores of years this same treatment has proved successful in eradicating this same disease from the system.

But the attorney's task is more difficult. Rarely has he the opportunity of an ex-parte trial, nor can he make use of evidence obtained from intuition, induction or demonstration; nor can he, in the great majority of cases, intrust his case to the judicial crucible with any surety of having their verdict influenced by the light of science.

The true question in trials of fact is not whether it is possible that the testimony may be false; but whether there is sufficient probability of its truth—that is, whether the facts are shown by competent and satisfactory evidence. The general rule in judicial tribunals is that the witness must confine himself to facts relevant to the issue joined and leave the conclusions from those facts to be determined by the Court or jury under oath. This is so when the case is plain. This is so when there are no long links in the chain of testimony, when eye-witnesses and ear-witnesses are available to show every detail. But frequently in the journey of proof there are broad aestuaries to span without piers seen by the eye or sounded to the ear, on which to rest the bridge of proof; and this is the emergency in judicial investigations where the medical expert is most needed. For instance H. is being tried for poisoning W. In H's possession is found a remnant of arsenic, accompanied by other suspicious circumstances proven by the State. The question will be whether W. was poisoned by arsenic. The fact that other persons who were poisoned with arsenic exhibited certain symptoms which experts affirm or deny to be the symptoms of that poison is deemed relevant.

Extra knowledge on any question of science, skill, trade, business or other matters requiring special knowledge, qualifies the person thus informed to give opinions in Courts of justice—contrary to the general rule that witnesses must confine themselves to the facts and leave the conclusions from those facts to be determined by the court or jury.

An opinion is a judgment which the mind forms on any proposition, statement, theory or event, the truth or falsehood of which is supported by a degree of evidence that renders it probable, but does not constitute absolute knowledge, truth or certainty. These opinions or conclusions of judgment which make up such opinions of experts are the same in substance as the verdict of a jury or judgment of a court, which is nothing more than the opinion of such jury or court as to what is established by the facts in the case. This conclusion or opinion in the latter case is given under the sanction of an oath. So is that of an expert,—with this difference in the two cases:—the court or jury is under oath while they are making up their opinions upon the facts in the case, and these facts, upon which the opinion is predicated, are also submitted to the minds of council and parties; the facts are also given by the common witnesses under oath, upon which the jury or court makes up an opinion. The expert, on the other hand, comes to the result constituting his opinion which is to be received in evidence, from his own private study, experience, observation and reflection,—the private judgment of a witness given under oath.

Of all the learned professions we find most frequently physicians deposing as experts on the witness stand, but in spite of this fact many members of the profession are inclined to look upon medico-legal practice as an unneccessary addition to the ordinary. But few of any repute practice long before they find themselves in situations of difficulty from some accidental occurrence of cases demanding medico-legal investigation:—

A medical man is called to attend A, who is suffering from a wound. In spite of best treatment death ensues. In his dying statement about the cause of his pending dissolution he accuses B. of stabbing him with a knife. At the death of A. the functions of a medical man end and those of a medical witness begin. The doctor can't escape giving evidence. He is summoned before the Coroner to describe the nature of the wound which caused the death, and there deposes that the wound was inflicted by some blunt instrument such as a club. But in spite of this opinion, the dying declaration goes to the grand jury, an indictment is found, and upon the trial of B. for murder the same doctor is again called to iterate his previous testimony to impeach a part and thus weaken the whole of A's dying declaration. He must there be prepared to answer numerous questions all bearing upon the legal proof of crime,—by what means this particular wound was inflicted, the direction , size and nature of the wound, with other circumstances tending to show accidental, homicidal, or suicidal means,—whether any and what statements were made by the dying man, the exact physical condition and surrounding circumstances under which those statements were made; for instance, whether the deceased had expressed hope of life at the time of or after said declaration.

The professional relationship between physician and patient gives rise to many emergencies on the criminal as well as civil side of the courts in which the physician must go upon the witness stand, regret it however much he may. No position is perhaps more embarrassing to a physician than that of an expert witness to whom partisanship is imputed by adverse counsel, but there is no alternative than to obey the subpoena in criminal cases, and always in this State in civil cases where the State is not a party he must obey the subpoena notify the officer at the time of service of subpoena that the witness is a practicing physician and claims the professional exemption from personal attendance on the judicial tribunal,—a return by the officer to which effect will be notice to the party to resort to his statutory alternative of taking the physician's deposition at his office. There may be a question as to whether the privilege must be claimed at the time of service of subpoena to exempt the physician from personal attendance. It is always best to do so in order to escape possible annoyance of a forfeiture for non-appearance and attendant attachment by which attempt would be made to bring a physician in. If he is not a material witness to facts, however, it will be impossible for the party needing his presence, truthfully to make such an affidavit as will warrant the issuance of an attachment. And even if he is a witness,—not simply an expert,—the forfeiture will be set aside by proper application to the Court showing that it was taken against a physician in active practice.

Many think it a great hardship to force medical men in courts to attend from time to time for the small pittance of one dollar per day. A subscriber to the Medical Magazine of the University of Pennsylvania, has written to the editor of that paper for information in regard to the power of a court to compel the attendance of medical men, with no compensation for their time and technical knowledge other than that ordinarily awarded to witnesses.

The question, by reason of its interest, was referred to an eminent member of the Philadelphia Bar, who replied that such a question may occur to a Doctor of Medicine:—First, where he will be called upon merely to testify as to the facts within his knowledge. Secondly,—when he has knowledge of medical facts in regard to a particular case and is called upon to state these facts and to give his professional opinion upon them. Thirdly,—Where he has no knowledge of the facts and is called upon merely to give a professional opinion.

Contrary to the law of Pennsylvania, in neither of these three cases would the physician be compelled to go personally into a Tennessee Court. He can be forced, however, to depose at his office in either of the first two instances for fees usually given to witnesses. In the third case he may claim extra compensation on the ground that the professional knowledge of a doctor is his property which he cannot be compelled to part with without compensation.

One will rarely force on the stand an expert purely as such without first hearing his opinion on the particular subject, and if this opinion is sought and request of attendance made either by the party, his agent or attorney before service of subpoena or between said service and trial, an implied promise of payment arises for professional services rendered, as plainly as when that promise arises upon a call to the litigant's bedside. Certain States have passed laws allowing extra compensation for experts to be fixed by the trial court.

To be an expert for the purpose of any trial may not be considered as a great honor; but it is the prerogative of the trial judge to decide, subject to the opinion of the Court above, whether the skill of any person in the matter on which evidence of his opinion is offered, is sufficient to entitle him to be considered as an *expert.*

So the profession can easily see that it is by no means impertinent on the part of council introducing or crossing him to inquire into the amount of experience the deponent has had, or to know the peculiar advantages he has had for the acquisition of knowledge. After qualification as expert the witness deposes his opinions simply. Posing as an expert, he is supposed to possess an opinion of his own and not that of another. He cannot read authorities on the stand in corroboration of any opinion he may assert. The attorney may read such authority on the subject at bar approved by the expert and conceded by the court to be a standard work on the subject. On cross-examination the physician should not equivocate or evade, but answer to the point, be the effect what it will, and although he cannot read authorities, he may expect on cross-examination to be interrogated upon the foundation of his opinion, may have literature quoted to him of his own profession, and be required to agree with or dissent from the opinion of the author quoted; whether he is familiar with certain works of that science, or not, and even with the latest edition. The witness must not do the objecting to questions but leave his protection to the counsel calling him. The judge will make him tell anything relevant, first, to his qualification and then to the issue,—disclose his age even unless there are special reasons for concealing it. If he is giving evidence for the State against a supposed poisoner in his trial for murder and is asked by the defense what remedy or antidote he had employed when he was first called to attend the deceased, an eminent judge has ruled that the physician shall answer unless he believes his antidote killed the deceased, in which case he would not be forced to criminate himself.

The futile attempt is sometimes made to shield professional communications between physician and patient, but to no avail. The law does not shield these confidences as privileged, and it were well for the physician not to encourage or invite any confidences the relation of which might criminate the patient.

A great fault with many experts in medicine is an attempt to display too much erudition, technicalities of the profession in which they are skilled,—a practice to be discouraged, as nothing is more necessary to the comprehension of a jury of ordinary men and nothing more convincing to a court and bar of a physician's skill in his profession than his ability to deliver himself in plain, untechnical Anglo-Saxon. Nothing is more pedantic than professional lingo on the witness stand by a man who demeans himself as if he were addressing a report to some president and members of a medical society instead of to a jury of unsophisticated men.

It will be asked whether a physician should prepare for any particular expert examination. Surely the party calling him will acquaint the expert with the nature of the case if the expert has had no previous connection with it.

To this we say, it will depend upon the expert's familiarity with the subject in the abstract. By far the most numerous occasions for this kind of evidence will be where the physician has had to do with one of the litigants in a professional capacity. In this last case there is a tendency in the expert to tell all he knows regardless of the *relevancy* of all or any part of it to the decision of the issue, and the expert is provoked at being stopped in his story. To illustrate:—A is being prosecuted for murder. He pleads insanity, is examined by his old family physician who has treated him through spells of sickness prior to the killing,—which physician is in turn put upon the stand as an expert. The question here is whether A. at the time of the killing was by reason of unsoundness of mind incapable of knowing the nature of the act or that he was doing what was wrong or contrary to law. The opinion of the doctor upon the question whether the symptoms exhibited by A. commonly show unsoundnes of mind, and whether such unsoundness of mind usually renders persons incapable of knowing the nature of the acts which they do, or of knowing that what they do is either wrong or contrary to law,—will be relevant to the issue. But it will be hard to convince the expert who has testified in that capacity alone that he is not to go into detail about the curious action of this man years ago. The expert will do well to listen to the witnesses as they depose to the symptoms, which symptoms will be incorporated into a hypothetical case by counsel to be propounded to the expert for his opinion.

In this connection will be mentioned what evidence will be relevant in the trial of malpractice suits against physicians.

Every person, lawyer, doctor or other professional man who offers his services to the public generally, impliedly contracts with employers that he is in possession of the necessary skill and experience which is possessed ordinarily by those who practice or profess to understand the same art or science and which is generally regarded by those most conversant with the profession or employment as necessary to qualify him to engage in such business successfully. Mr. Justice Story says:

—"In all those cases where skill is required it is to be understood that it is to be ordinary skill in the business or employment which the bailee undertakes, for he is not presumed to engage for extraordinary skill which belongs to a few men only in their business or employment,—nor for extraordinary endowments or acquirements. Reasonable skill constitutes the measure of the engagement in regard to the thing undertaken.

The common law rule is that every one who enters into a learned profession undertakes to bring to the exercise of it a reasonable, fair and competent degree of skill. The nature of the implied contract between the physician called and patient calling him is not to warrant a successful operation or insure a cure; but if he does make an express contract for absolute cure the utmost diligence and skill will not excuse him should the result be unfortunate after taking pay on such conditions, and he will further be liable upon a malpractice suit for damages in the nature of a breach of warranty.

But, in defense of such suit it will be relevant to show by experts that the undertaking was impossible, in which case the law will not hold him responsible for the full extent of the damage resultiug to the patient by reason of his failure after using all skill. He will simply forfeit all compensation for medicine and service.

Where there is no express contract and the treatment is a failure, at trial the question to plaintiff's expert is not whether the expert could have effected a cure, or whether ordinary skill could have done so, but whether the care and skill of the defendant's treatment, as deposed by witnesses, was ordinary care and skill.

When the expert deposes intelligently, keeps his head, and shows no partisan feeling he can hardly realize the weight that is given to his testimony. A noted writer on evidence has classified the grounds of belief in human testimony as follows:—

1.—The instinctive principle of credulity implanted in man's nature.

2.—The general experience of the truth of human testimony.

3.—The known and experienced connexion between collateral facts satisfactorily proven and the facts in controversy.

Under this last heading falls all of expert testimony. Mr. Paley describes the general character of human testimony as:—"Substantial truth under circumstantial variation." No two witnesses will rarely describe the same occurrence exactly alike. There will always be some variation, attributable, it may be to the eye of the beholder, or ear of the hearer, as much as to the thing seen or heard.

Law is by some of the learned professions criticised as a retrospective science. But, however true may be this imputation in one respect, it is making strides. It is no longer so necessary as formerly that any eye-witness should see, or ear-witness should hear the crime being committed in order to a conviction. Say all modern courts and the verdict of modern juries that a man may involve himself in a net-work of circumstantial evidence less easy to impeach than the evidence of one single witness testifying to his actual perception by the senses. Persons may lie; science never.

But boundaries must be fixed to the introduction of such evidence and it is essentinel to circumstantial proof, be it deposed by medical experts or others:—

First:—That the circumstances from which the conclusion is drawn should be fully established.

Second:—That all the facts proved should be consistent with the hypothesis.

Third:—That the circumstances should be of a conclusive nature and tendency.

Fourth:—That the circumstances should, to a moral certainty actually exclude every hypothesis but the one propounded to be proved.

Fifth:—That mere circumstantial evidence, unless the chain of circumstances is absolutely complete, ought in no case to be relied on where direct and positive evidence, which might have been given, is withheld by the adverse party.

Read before the Tri-State Medical Association, in session at Chattanooga 14 Oct. 1890.

